# Effect of repeated intakes of a neonicotinoid insecticide on the foraging behaviours of *Apis mellifera* in field trials

**DOI:** 10.1007/s11356-022-22977-y

**Published:** 2022-09-15

**Authors:** Vincenzo Girolami, Edoardo Petrucco Toffolo, Luca Mazzon, Francesca Zampieri, Andrea Lentola, Chiara Giorio, Andrea Tapparo

**Affiliations:** 1 Dipartimento di Agronomia Animali Alimenti Risorse Naturali e Ambiente, Università degli Studi di Padova, viale dell’Università 16, Legnaro, Padova, 35020 Italy; 2 Dipartimento di Scienze Chimiche, Università degli Studi di Padova, via Marzolo 1, Padova, 35131 Italy; 3 Laimburg Research Centre, Laimburg 6, 39040 Ora, Bolzano, Italy; 4 Yusuf Hamied Department of Chemistry, University of Cambridge, Lensfield Road, Cambridge, CB2 1EW UK

**Keywords:** Honeybee behaviour, Foraging activity, Clothianidin toxicity, Sub-lethal doses

## Abstract

Evaluating the effects of neonicotinoids on forager bees in conditions as near as possible to those in nature presents a considerable challenge. Tackling this challenge is, however, necessary to establish their negative side effects on these pollinators. For instance, it is still under debate the mechanism by which bees seem to recognize low-level contaminations of neonicotinoid insecticides in nectar and pollen of the flowers they visit and limit collection to protect themselves and their hive from a possible intoxication. In this study, we propose an experimental system that involves the use of foragers in free flight foraging repeatedly on artificial feeders containing a sucrose solution contaminated with clothianidin, as well as foragers feeding at adjacent control feeders, allowing us to observe changes in their foraging activity. The progressive disappearance of foragers from the contaminated feeders became increasingly clear and rapid with the increase in clothianidin concentration. The lowest concentration at which we observed an effect was around 10 µg/L, which corresponds to the maximum residual concentration (10 ng/g) observed in pollen and nectar of flowers close to open fields sown with seeds coated with insecticides. At the highest concentrations tested (80 µg/L), there was an almost total abandonment of the feeders. The estimated quantity of contaminated sucrose solution collected by foragers showed an almost linear relationship inversely proportional to clothianidin concentration, whilst the estimated quantity of insecticide collected by a forager increased and then stabilised at the highest concentrations tested of 40 and 80 µg/L. Irregular mortality was not observed in front of the hives, furthermore, foragers did not show evident memory of the position of the treated units in the trials on the 2 consecutive days. The decrease in foraging activity in the presence of a few µg/L of insecticide in the sucrose solution appears to limit the introduction of elevated amounts of toxic substances into the hives, which would have serious consequences for the young bees and the brood. At the same time, in the absence of an alternative energy source, even reduced feeding of the hive can compromise colony health.

## Introduction

The bee population has shown a worldwide decline in recent years (Williams et al. 2010; Wood et al. 2020). The identified causes of such decline were pests, diseases, pesticides, loss of forage, and not suitable beekeeping practices (Stankus 2008; vanEngelsdorp et al. 2009). The specific symptoms and the seasons in which the decline occurs may help in recognising the causing factors; for example, *Varroa destructor* plays a decisive role during the winter as confirmed in Germany (Genersch et al. 2010) and North America (Guzmán-Novoa et al. 2010). Mortality may also be due to exposure to neonicotinoids, which are among the most widely used insecticides in the world (van der Sluijs et al. 2013). In France, the first suspicion that bee deaths may be caused by the use of neonicotinoids was raised when observed mortality coincided with the flowering of sunflowers from plants treated with imidacloprid. This led to the ban on the use of sunflower seeds coated with imidacloprid in 1999 and subsequent extensions to the ban (Maxim and van der Sluijs 2007). In Italy, bee mortality coincided with the sowing of maize; this led to a ban on maize seeds coated with the neonicotinoids imidacloprid, clothianidin, and thiamethoxam, together with the phenylpyrazole fipronil in 2008, and subsequent extensions. After a first ban (2013), in 2018 the European Commission imposed a definitive moratorium on the use of seeds coated with neonicotinoids (European Commission 2018) (earlier for fipronil (European Commission 2016)) on bee-attractive crops. Despite this, neonicotinoids are the most widely used insecticides in the world, being employed in more than 120 countries and on 450 crops (Simon-Delso et al. 2015; Bakker et al. 2020).

In the case of maize crop, the foragers in free flight can come into contact with particles of seed coating containing the insecticide emitted by pneumatic seed drillers (Girolami et al. 2012; Tapparo et al. 2012) or when foraging on flowers at the field margins (Greatti et al. 2003). Given the neurotoxic properties of neonicotinoids (they are agonists of the nicotinic acetylcholine receptors of the insects), the foragers can die after having flown through a cloud of seed coating dust as a result of the high exposure doses to the insecticide, in the orders of hundreds of ng/bee, sufficient to cause acute poisoning (Girolami et al. 2012). By contrast, the concentration of neonicotinoids in pollen and nectar from plants germinated from coated seeds or flowers at the field margins is generally below 10 ng/g (Bonmatin et al. 2003; Rortais et al. 2005; Cresswell 2011; Long and Krupke 2016). Such contamination in flowers could not lead to acute poisoning of foragers but these sub-lethal doses can impact learning, performance, behaviour, and neurophysiology (Desneux et al. 2007; Blacquière et al. 2012).

Foragers carry pollen in their pollen baskets on their hind legs. Such baskets have no connection to the digestive tract; contaminated nectar, taken orally and retained in an enlargement of the oesophagus called honey stomach, could be absorbed with immediate consequences. Evaluating the effects of contaminated nectar intake on foragers in the field is a key issue. These experiments are problematic due to the difficulties of studying the fate of free-ranging foragers and the colony (Henry et al. 2012). In fact, some factors can influence metabolism and can have consequences on insecticide ingestion. For instance, bees can defecate only in flight and, besides, foragers return to the hive rapidly to regurgitate the collected nectar. Yet another aspect concerns communication through “the dance”, which may play a decisive role in the use of food sources (Kietzman and Visscher 2015) and can influence the behaviour of foragers visiting those contaminated with insecticides (Cox and Wilson 1984; Kirchner 1999; Fischer et al. 2014).

A vast review of results obtained in field or semi-field conditions about the behavioural effects of pesticides in bees is reported in Thompson (2003). Initially, the trials evaluated the effects of insecticides on free-ranging foragers making repeated flights over treated plots (Anderson and Atkins 1958; Shires et al. 1984; Gary and Lorenzen 1989), or flights to contaminated feeders (Cox and Wilson 1984; Schmuck 1999; Waller et al. 1979). Organophosphate and pyrethroid insecticides were causing a reduction in the activity of foragers (Waller et al. 1979; Shires et al. 1984; Gary and Lorenzen 1989) and bee deaths at higher concentrations (Anderson and Atkins 1958). Schmuck (1999), the first to study neonicotinoids and foragers, observed a decline in the visits to artificial feeders at a concentration of 100 ppb of imidacloprid; effects were also confirmed in semi-field trials (Ramirez-Romero et al. 2005; Decourtye et al. 2004). Recent studies have focused on the effects of offering a single dose of poisoned sucrose solution either on foragers kept in captivity for a pre-determined time (Bortolotti et al. 2003; Schneider et al. 2012) or free from any captivity (Yang et al. 2008).

In a 2010 review by Decourtye and Devillers (Decourtye and Devillers 2010) considerable variations in the toxicity of neonicotinoids ingested in laboratory trials were observed, which were attributed to physiological and behavioural differences of the bees and different experimental methods used.

In this work, we have studied the effects of clothianidin, one of the most widely used neonicotinoids, on *Apis mellifera* foragers free to make repeated flights to contaminated feeders in field trials at conditions as near as possible to natural conditions of foraging. The missing foragers after visiting the artificial feeders treated at different concentrations of clothianidin were evaluated in a synchronous comparison with adjacent control feeders.

## Materials and methods

Experiments were performed on colonies of *Apis mellifera* (hybrid ligustica and carnica) located on the experimental farm of the University of Padua (Legnaro, 8 m above sea level; 45.3451° N, 11.9541° E) during 2 successive years (in spring–autumn). The honeybee colonies were replaced every spring with new nuclei. The hives did not receive chemical treatments during the period of the experiments and were periodically opened to check the presence of the queen and brood and to evaluate the state of health. Underbaskets (as described in Accorti et al. (1991)) were placed on the ground in front of the hives to evaluate abnormal mortalities. Other hives were present in the surrounding area at a distance of at least 500 m.

### Chemicals

A stock solution of 100 mg/L of clothianidin (from the pure compound, PESTANAL analytical standard, SigmaAlrich, St Louis Missouri, USA.) was prepared in ultrapure water (purified with a Millipore Milli-Q® equipment) and kept in a refrigerator at 4 °C. On the day of the trials, the stock solution was diluted with a sucrose solution (5–20% w/w) to the required concentrations (see “Experimental procedure”). Neither solvents nor scents were added to the solutions.

### Feeder setup

In the first-year tests, the feeders consisted of a petri dish on which a wire net (0.5 mm mesh) was fixed to allow for the landing of foragers; such feeders were placed on the ground of an open field, without crops.

**Fig. 1:**
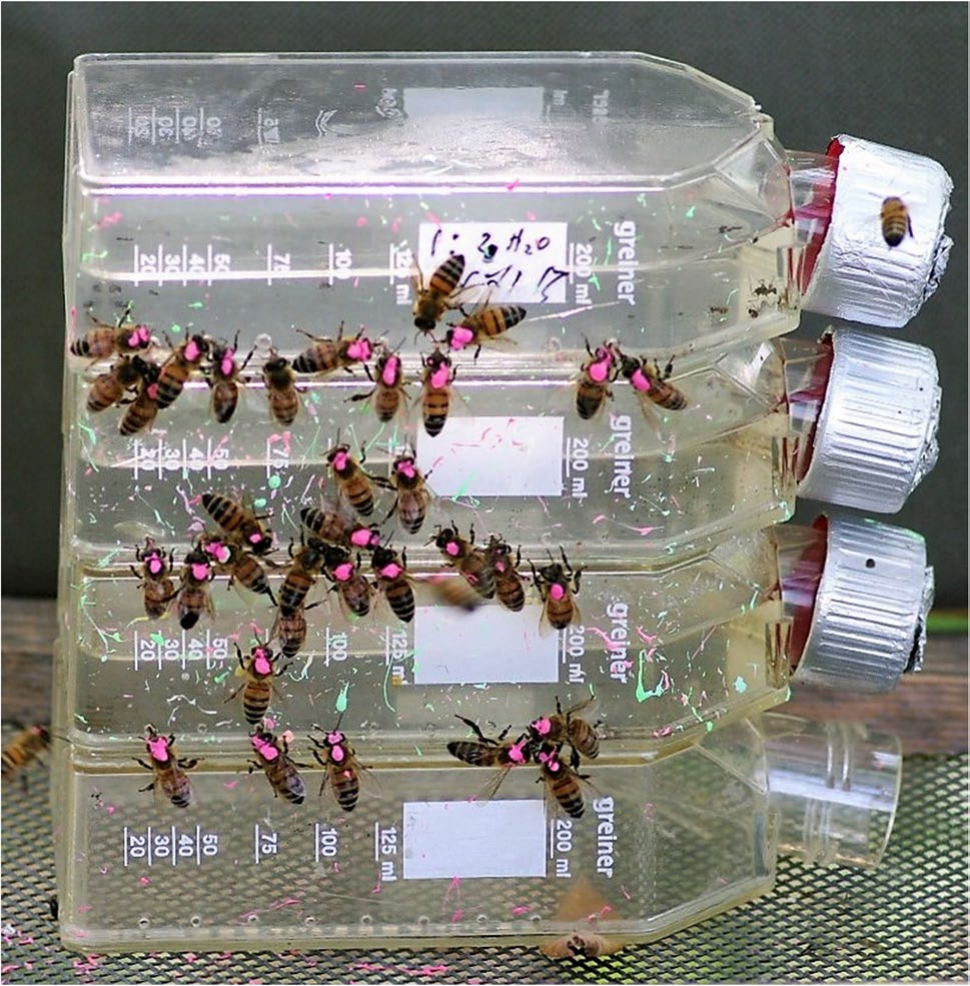
Sucrose solution feeder optimized for the counting of the attracted forager bees

To increase the repeatability of the results, a new type of feeder was used in the second-year experiments. It consisted of a stack of three flasks (standard cell line culture flasks 250 mL VWR®) with nine holes (3 mm in diameter) at the base of a lateral surface drilled 1 cm from each other. A fourth flask was used as a support. Each flask was filled with 150 mL of a sucrose solution (5–20% w/w, containing a defined concentration of clothianidin in the range of 2.5–80 µg/L). The three flasks were placed one above the other to allow for photographic counting of the foragers on a single vertical surface. This stack also allows for their markings with a brush or a folded wire (Fig. 1). A rigid wire net was placed at the base of the three feeders; this precaution avoided the accumulation of leakages of sucrose solution that, upon evaporating, could increase sucrose and insecticide concentrations, with the consequent greater attraction of the foragers and their exposure to the insecticide. At the base of the structure, 1 m high, supporting the wire net, a tangle foot ring was placed to inhibit ants, which could disturb the bees.

### Experimental procedure

At the beginning of each trial, the feeders without the insecticide were placed close to the hive to allow foragers to get used to visiting them; then, they were gradually moved towards the experimental site that was about 30 m far from the hive. A single petri dish in the first year and the three flasks, stacked one on top of the other in the second year, constituted the feeding unit (or feeder). Once the foragers had been trained to fly to the experimental site, six feeding units were placed on a line equidistant from the hives and separated 2 m from each other. The foragers distributed themselves, spontaneously, in similar numbers on the six feeders. This set was maintained for some days allowing the foragers to memorize the place of the sucrose feeders.

On the appointed day, the trials started at about 10 am, time in which all feeders were filled with sucrose solutions to attract the foragers. When the flight was fully established (after about an hour), the foragers visiting the feeders were counted every 15–20 min by visual and photographic analysis. The pre-treatment phase without insecticide in all the units lasted for 60 min. At the end of this first phase, according to a scheme established a priori, three feeding units were filled with a plain sucrose solution (control units) and three feeding units were filled with the identical sucrose solution containing the insecticide (treated units). The treated units (T) and control units (C) were placed with two treated units and two control units at the two extremes, alternating in the middle (TT C T CC).

The feeders remained available to the bees for 160–165 min. In some trials (with clothianidin concentrations of 20 and 40 µg/L) a longer treatment phase (200 min) was tested to verify the robustness of the procedure. To obtain a number of foragers of about 15 to 45 per feeding unit, the concentration of the sucrose solution varied from 5 to 20% (w/w) depending on the environmental conditions. In effect, the presence or lack of competitive flowering influenced the average number of bees visiting the dispensers. If the number of foragers in the pre-treatment phase fell below a value of 12 per feeder, the test was repeated using a sucrose solution at higher concentration.

### Foragers missing and clothianidin concentration

To assess the dose–effect relationship, in the first-year experiments we tested the clothianidin concentrations of 10, 20, 40, and 80 µg/L, extending the exposure range to lower concentrations (2.5, 5, 10, 20, 40, and 80 µg/L) in the second year. The number of trials for each concentration level (but carried out on different days) increased from two to three trials in the first and second year, respectively.

To verify possible memory effects toward forager bees, some trials (20–40 µg/L) were repeated on 2 consecutive days reversing the positions of the three control and the three treated units with respect to the previous day.

### Quantity of solution and insecticide collected by bees

The availability of a series of data on the relationship between the clothianidin concentration in sucrose solutions and the number of foragers visiting the feeders allowed the estimation of the average number of bouts and quantity collected from a single forager during each treatment phase. The average amount of sucrose solution and insecticide collected by a forager (Table 1) was assessed at different concentrations of insecticide using parameters found in the literature, coming from observations on artificial feeders in the field and confirmed in the present study. The average time employed by a forager to make a bout (220 s, as the interval between two successive flights) and to intake the solution from the feeder (90 s) were both taken from Schneider et al. (2012). According to Yang et al. (2008) and von Frisch (1967), it was considered that 50 µL is the average quantity of solution collected by a forager in one visit.

**Table 1:** Estimated average values of the number of flights, intake of sucrose solution and of insecticide per forager visiting the feeders at different concentrations of clothianidin

Clothianidin concentration (µg/L)	Number of flights	Intake of sucrose solution (mL)	Insecticide intake (ng)
5	36.5	1.83	9.13
10	34.8	1.74	17.4
20	26.4	1.34	26.7
40	17.6	0.88	35.2
80	9.0	0.45	36.0

Besides the foragers photographed at the feeders, at the same time there were others in flight or inside the hive. The total number of foragers involved in foraging (*N*_*foragers*_) was obtained by multiplying the average number of foragers counted (photographically) during the pre-treatment phase (*N*_*pre-treat*_) by the relationship between the time employed to complete a bout (*t*_*bout*_) and the time necessary for solution intake (*t*_*intake*_) at the feeder (Eq. (1)).

1 $${N}_{foragers}={N}_{pre-treat}\frac{{t}_{bout}}{{t}_{intake}}$$

For each insecticide concentration, we calculated the area under the interpolated response curves of N_foragers_ vs time (f(t)) between the administration of the insecticide (60 min) and the end of the observations (225 min), with a Δt corresponding to the intake time on the feeder (90 s). This value corresponds to the total number of visits and thus the total number of bouts (N_bouts_) of the foragers (Eq. (2)).

2 $${N}_{bouts}={\int }_{60\mathrm{min}}^{225\mathrm{min}}f(t)\Delta t$$

The average number of bouts made by a forager (N_bouts/forager_) during the treatment phase (Eq. (3)) was obtained by dividing the total number of bouts (N_bouts_ from Eq. (2)) by the number of foragers involved (N_foragers_ from Eq. (1)).

3 $${N}_{bouts/forager}=\frac{{N}_{bouts}}{{N}_{foragers}}$$

The average quantity of sucrose solution collected per forager (V_collected_ in mL) during the treatment phase (Eq. (4)) can be obtained by multiplying the average number of bouts per forager (N_bouts/forager_) per 50 µL of solution ingested (V_ingested_ in μL) in one bout.

4 $${V}_{collected}={N}_{bouts/forager}{V}_{ingested}{10}^{-3}$$

If the quantity of solution collected by a single forager (V_collected_) is multiplied by the respective concentration of insecticide (C in µg/L), it is possible to estimate the average dose of insecticide collected per forager (D_forager_ in ng, Eq. (5)).

5 $${D}_{forager}={V}_{collected}C$$

### Statistical analysis

A non-parametric kernel regression was used to examine the time dependence of the visiting patterns of the foragers and to compare trends in control and treated units. Moreover, we analysed the variations in the number of foragers exposed to each concentration of insecticide in successive observations. For each concentration, we used a probit analysis to establish the time in which the halving of the population of foragers visiting the units occurred. Subsequently, we used linear regression to analyse the relationship between clothianidin concentrations and halving time, having three independent values for each concentration. The kernel analysis was performed with *r*, and the probit analysis with xlstat software.

## Results

### Missing foragers and clothianidin concentration at the feeder

**Fig. 2:**
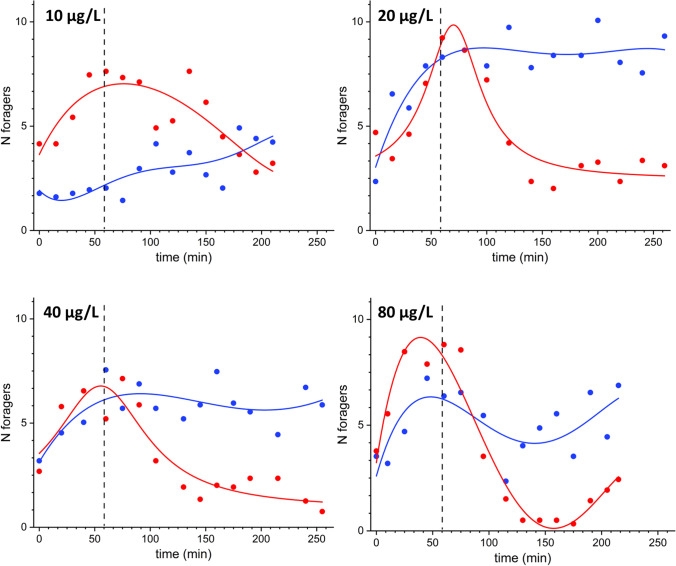
Variation of the average number of foragers vs. time on control and treated feeders (petri dishes) containing sucrose solutions at different clothianidin concentrations: 10–20–40–80 µg/L. The dots represent the average of two independent observations of experiments carried out on different days; the lines are the result of non-parametric kernel regression analysis. Blue: control units; red: treated units. The vertical dashed line indicates the beginning of the treatment phase

**Fig. 3:**
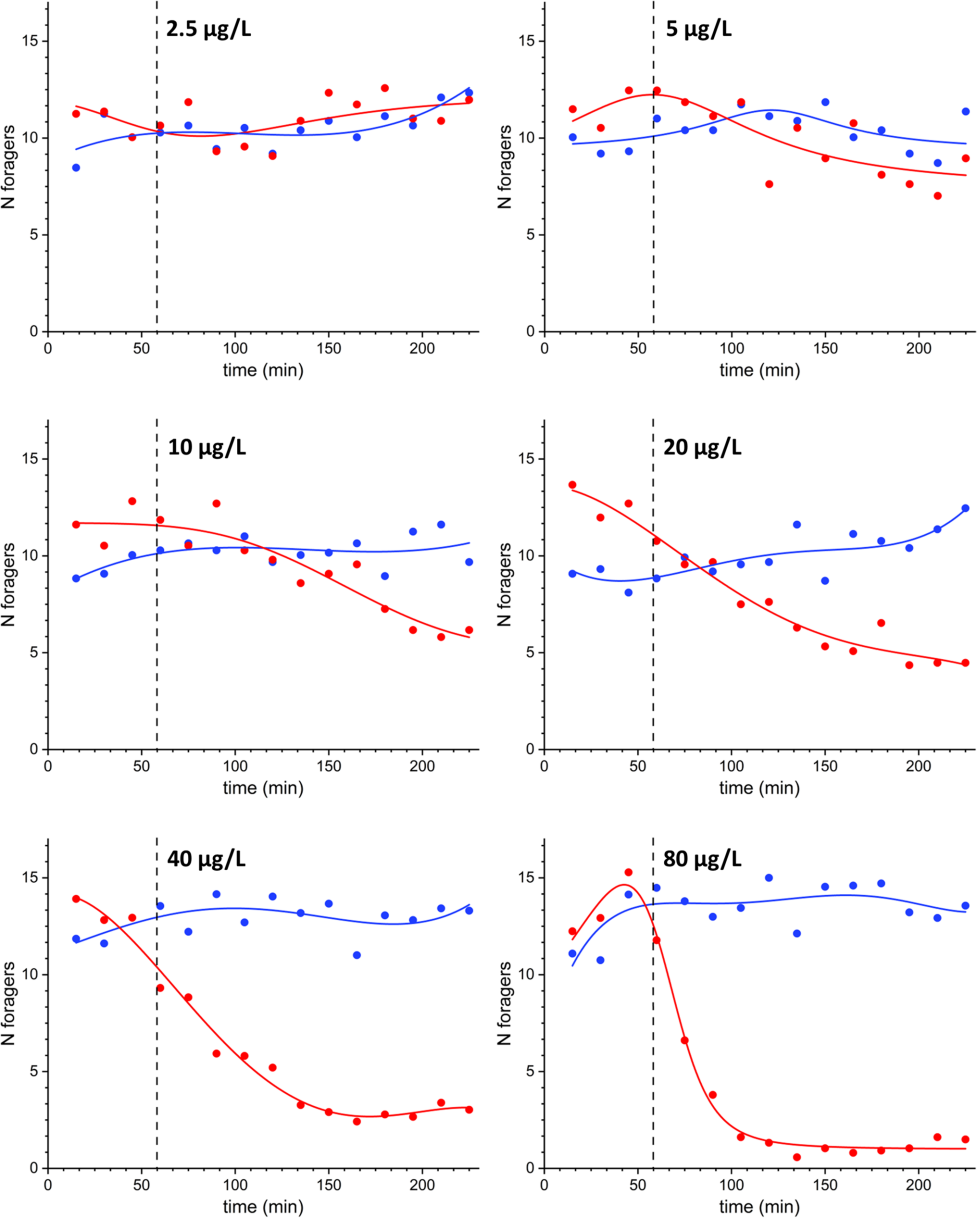
Variation of the average number of foragers vs. time on control and treated feeders (staked flasks, Fig. 1) containing sucrose solutions at different clothianidin concentrations: 2.5–5–10–20–40–80 µg/L. The dots represent the average of three independent observations of experiments carried out on different days; the lines are the result of non-parametric kernel regression analysis. Blue: non-treated (control) units; red: treated units. The vertical dashed line indicates the beginning of the treatment phase

The reduction in the number of foragers visiting the feeders of treated units showed a clear dose–effect relationship with the concentration of clothianidin. During the treatment phase, we observed a gradual reduction in the visits by the foragers (Figs. 2 and 3). The number of foragers visiting the control units, at the same time, showed no substantial variation with respect to the values in the pre-treatment phase, maintaining, in the different trials, a higher value compared to the treated units. There was no evidence of an increase in numbers in the control units, despite the visible decrease in the contiguous treated units. The results obtained in the trials of both years (using two different feeders, Figs. 2 and 3) showed similar trends, but in the latter case the uncertainty (variability) in the number of foragers was significantly lower, revealing the improving performance of the stacked flask feeder. For all the tested concentrations, kernel analysis showed a statistically significant reduction of the number of foragers visiting the contaminated feeders (*p* < 0.001) except for the 2.5 µg/L concentration level (*p* = 0.08). At a concentration of 5 µg/L, there was a slight decrease in visits when compared to the control (Fig. 3), even though there was a higher variability in the response among trials. The decrease was more consistent at 10 µg/L. At the concentration of 20 µg/L, the number of foragers showed an evident reduction after 50 min; at a concentration of 40 µg/L the decrease appeared already from the first checks. Finally, at 80 µg/L, the curve showed a trend like that at 40 µg/L but with a steeper slope and an almost total abandonment of the treated units.

**Fig. 4:**
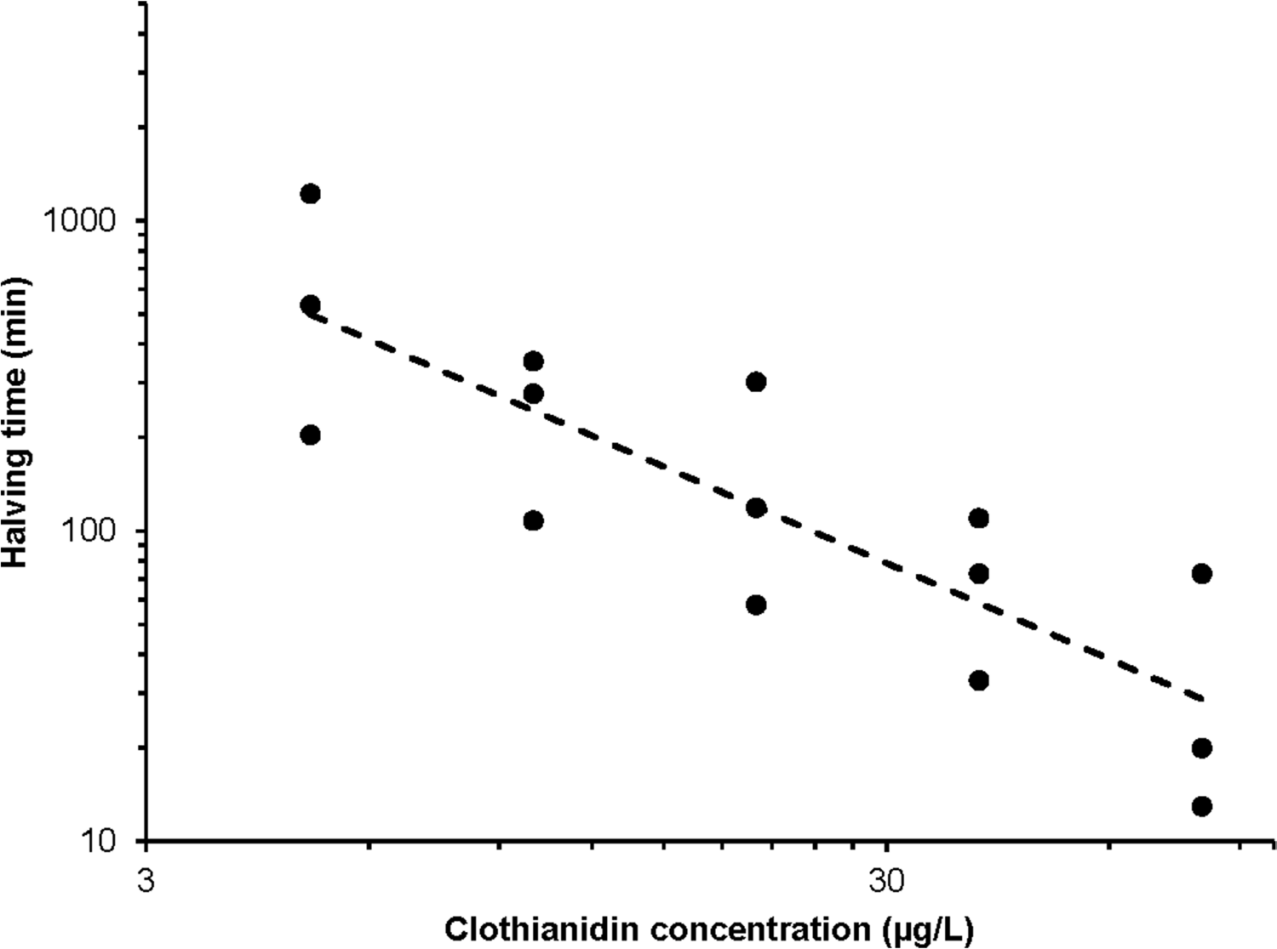
Relationship between clothianidin concentration and time of halving of the number of foragers on the feeders. Dots indicate the halving time of three independent trials at each concentration (log scale is used for both concentration and halving time)

The time in which half of the foragers disappeared from the treated units, resulting from the probit analysis, exceeded 165 min of the treatment phase at concentrations of 5 and 10 µg/L, whereas it was lower at 20 µg/L (Fig. 4). The halving time decreased with increasing concentrations reaching slightly over 1 h at a concentration of 40 µg/L and about half an hour at 80 µg/L. Using a logarithmic transformation of both variables (halving time vs clothianidin concentration, Fig. 4), a linear dependence with a significant negative slope is obtained.

**Fig. 5:**
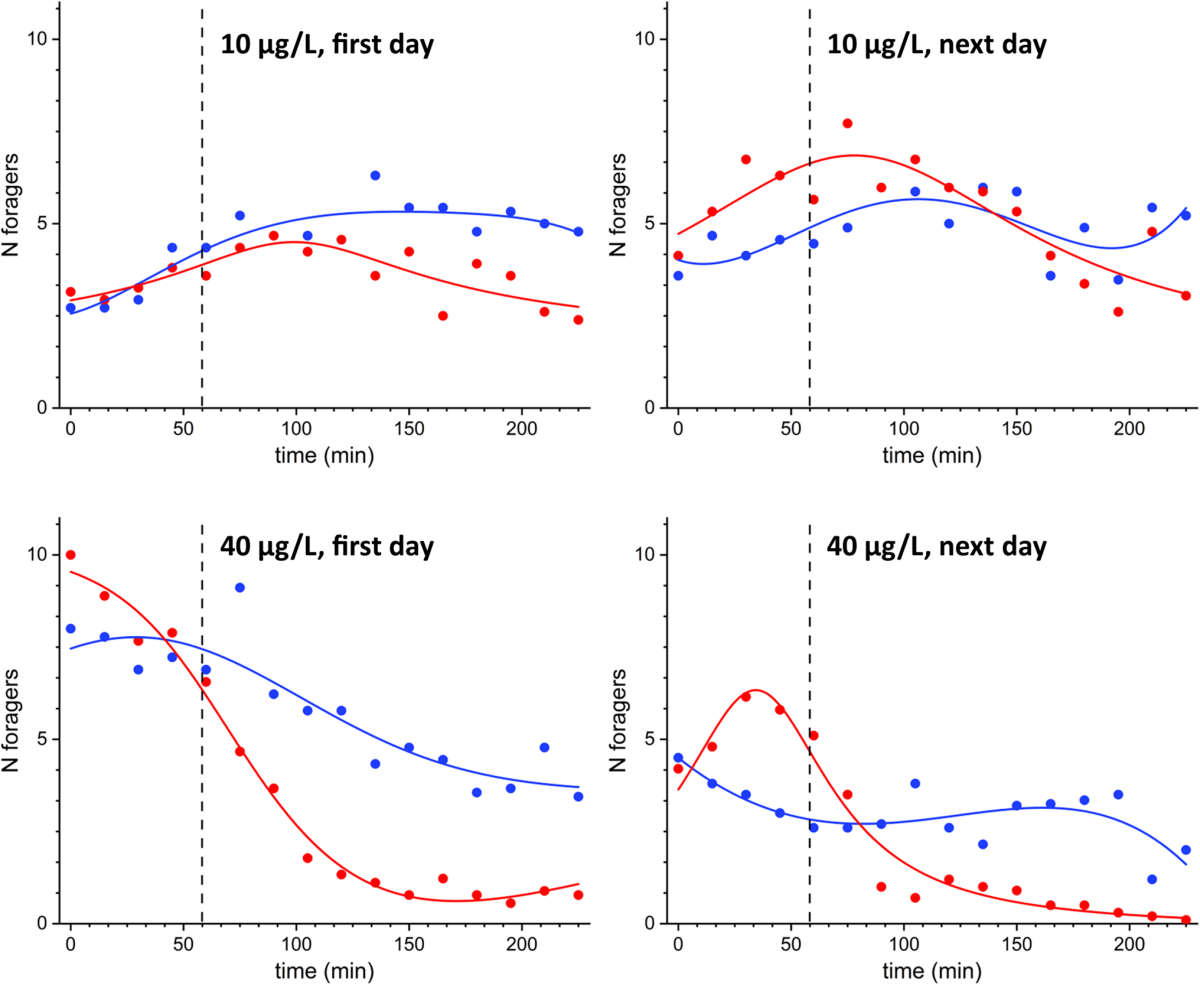
Variation of the average number of foragers (dots) vs. time observed on a single-day experiment on control and treated feeders containing sucrose solutions at 10 and 40 µg/L of clothianidin. Lines are the result of non-parametric kernel regression analysis. Blue: non-treated units; red: treated units. The graphs on the left refer to the first day of experimentation, and the graphs on the right report the results obtained in the experiment carried out on the subsequent day with the inversion of the position of the treated and untreated units. On the second day of the experimentation, the foragers re-colonized the treated units that had been abandoned on the previous day. The vertical dashed line indicates the beginning of the treatment phase

Reversing the position of control and treated units on consecutive days showed on the second day that the foragers re-colonized the treated units that had been abandoned the previous day (Fig. 5). The treated units saw a gradual decrease in the number of foragers, in a similar trend to that shown in Fig. 3. A statistically significant difference (*p* < 0.001) was found between control and treated units in all trials for both concentrations tested (10 and 40 µg/L).

### Amount of sucrose solution and insecticide collected per forager

As reported above, the average number of trips showed a decrease with increasing clothianidin concentration at the feeder; consequently, also the average quantity of sucrose solution collected per forager decreased (Table 1). At the lower concentrations tested, the two parameters showed similar values: 36.5 trips and 1.83 mL of solution collected at 5 µg/L; 34.8 trips, and 1.74 mL of solution collected at 10 µg/L. Significantly lower values were observed for sucrose solutions at higher concentrations of clothianidin. Remarkably, the average amount of clothianidin collected and transported to the hive, still referring to a single forager, showed an opposite tendency (Table 1): from a lower value estimated at the concentration of 5 µg/L (9.13 ng), there was a gradual increase to 35.2 ng estimated at the concentration of 40 µg/L with a similar value at the highest concentration tested of 80 µg/L (36.0 ng).

## Discussion

The experimental system adopted allowed the evaluation of the impact of ingestion of sucrose solutions with progressively increasing concentrations of clothianidin on the behaviour of forager bees visiting artificial feeders in free-flight in the field. The simultaneous survey of the control feeders, close to those treated (containing the insecticide), allowed for observing changes in behaviour due to varying environmental factors. The possibility offered to foragers of collecting sucrose solution with continuity approaches what happens in nature in the presence of inviting nectar sources or abundant production of honeydew. Besides, after a significant dose-related abandonment of contaminated feeders, the foragers do not seem to retain a memory of previous experiences with the insecticide, given that they were not biased by changes in the distribution of the treated and control feeders in trials carried out on consecutive days. On the other hand, the tests with tagged foragers revealed a surprising loyalty of foragers toward visited units on the same day of experimentation.

In the present study (both years), foragers from non-monitored hives were also observed on feeders in open fields and the battle, sometimes mortal, that took place at the units between competing colonies was resolved in favour of the foragers from the strongest hive, both at treated and control units. Nevertheless, despite the varying provenance of the foragers, the repetition of the trials in 2 years with numerous replications for each concentration has given uniform and comparable results. The presence of foragers belonging to non-monitored hives on treated feeders entails the uncontrolled transport of insecticide into some hives. Therefore, it is advisable to conduct experiments with isolated hives without other colonies in the area (Stanley et al. 2010), such as for example in Bortolotti et al. (2003).

The halving time of the number of foragers on the treated units became shorter with increasing concentrations of clothianidin and provided a good further indicator for the evaluation of the concentration effect (Fig. 4). The halving within a defined time, e.g., 1 or 2 h, could be useful in establishing the toxicity (more generally, the perception of the toxic substance by the bees), not only at different concentrations but also among substances. At the highest concentration tested (80 μg/L) the halving occurred in around 30 min. Although it was more rapid than that observed at lower concentrations, the abandonment of the treated feeder by foragers is not immediate, suggesting that these levels of insecticide in the sucrose solution do not stop or inhibit feeding rapidly. Most likely, these sub-lethal doses act slowly on the honeybees. The absence of an anti-feeding effect on foragers agrees with the results of laboratory trials carried out with imidacloprid (Decourtye et al. 2003). Furthermore, the intake of a single dose of 50 µg/L of clothianidin did not show to compromise the recently acquired memory, important for returning to the hive from a usual foraging site (Fischer et al. 2014).

The missing foragers on the treated units represent a clear change in behaviour with evident consequences in the energy supply to the hive. However, some of the foragers continued to visit the treated units for the whole duration of the trial, and they seemed less sensitive to the insecticide. This is an aspect which needs further investigation considering the complex division of labour among foragers (Biesmeijer and Vries 2003; Wagner et al. 2013).

### Behaviour on subsequent days

During trials performed on consecutive days, the foragers returned, in consistent numbers, to the control units which had been treated on the previous day (Fig. 5), as happened in the trials that used a single-dose intake (Schneider et al. 2012; Yang et al. 2008) with definite disappearances only at higher concentrations (Yang et al. 2008). The return of foragers on consecutive days indicates that, in general, at the tested concentrations, there was no prominent mortality, as suggested by the absence of abnormal accumulations of dead bees in the underbaskets placed in front of the hive. In our trials, we cannot exclude that a portion of the foragers may have died unobserved, and they may have been substituted by others. Further trials with tagged foragers would be necessary.

### Clothianidin concentration and dose–effect relationships

We witnessed around 90% of missing free-ranging foragers in repeated flights at the treated units, at concentrations of 80 µg/L of clothianidin (Fig. 3). Always in free flight and with repeated intakes at the contaminated feeders, a similar percentage of missing foragers was observed at 100 ppb (≈ µg/L) of imidacloprid (Schmuck 1999), considering the higher toxicity of clothianidin compared to imidacloprid (Waller et al. 1979). Always in free flight but with a single insecticide dose, around 90% of missing foragers occurred at 1600 ppb of imidacloprid (Yang et al. 2008). Therefore, a similar effect in repeated visits occurred at concentrations about 15 times lower with respect to a single dose. Repeated intakes likely cause a progressive accumulation of insecticide. In fact, the estimated cumulative dose with repeated intakes (Schmuck 1999) showed no significant difference with the dose administered with a single intake (Yang et al. 2008). Considering an average intake of 50 µL per trip, a single forager ingested an average quantity of 35 ng of clothianidin in our trials and 80 ng of imidacloprid in single-dose trials from Yang et al. (2008) showing in both cases around 90% of missing foragers.

Other studies observed a high percentage of missing foragers with single-dose intakes at concentrations much lower than 1600 ppb in Yang et al. (2008), for example at 150 ppb for clothianidin and 300 ppb for imidacloprid (Schneider et al. 2012), corresponding to 1 ng of clothianidin and 3 ng of imidacloprid ingested by a single bee. These studies, however, were performed with foragers kept in captivity both during and following the insecticide intake, thus impeding the rapid regurgitation of the sucrose solution in the hive and probably favouring diffusion of the insecticide into the insect body, as suggested by Bortolotti et al. (2003). An observed effect at a concentration lower than 100 ppb of imidacloprid (Bortolotti et al. 2003) could be due also to further metabolization of the solution caused by keeping the foragers in lighted cages.

In the field, analysing the ingested quantity of insecticide by the foragers is challenging; therefore, the main parameter used for drawing conclusions was the concentration. The maximum concentration encountered in pollen and nectar was around 10 ng/g (ppb) resulting from both the use of coated seeds and residues from previously treated crops. The minimum concentration in which we observed an effect in our tests on bee behaviour is around a similar value of 5–10 µg/L (ppb). This would indicate an intoxication at very low concentrations but with no evident mortality or missing bees (Fig. 3). This result may suggest the possibility that the bees could identify the presence of neonicotinoids (via specific toxic effects or via gustatory perception) thus avoiding their collection (Karahan et al. 2015). Alternative hypotheses could consider the signals communicated to the foragers by the bees in the hive which recognize low levels of insecticide in the collected food or a temporary disorientation of the foragers (Decourtye et al. 2011). Of course, the real foraging activity in the field, with flights longer than 30 m, may cause measurable effects at clothianidin concentrations lower than 5 µg/L.

## Conclusions

The experimental system adopted approaches natural conditions and is particularly sensitive to low insecticide concentration levels. In the first visits to the contaminated dispenser, the foragers did not seem to be alerted by the presence of a specific insecticide in their food or their bodies but following accumulation in repeated visits, a change in their behaviour with an interruption of foraging activity was observed. Similar behaviour was also reported with other insecticides such as organophosphates (Waller et al. 1979). At the concentrations tested, the missing foragers did not experience acute poisoning. So that the next day they tend to return to food sources without apparent memory of the contamination experienced on the previous day. Therefore, the presence in the field of insecticide residues at concentrations in the order of 10 ng/g (i.e., contaminated nectar or pollen) seems therefore unlikely to have caused the serious bee deaths that were encountered in Italy, Slovenia and Germany at the beginning of the 2000s, even though acute effects, with lethal outcomes at higher doses, were observed when bees were exposed to contaminated guttations and seed coating dust (Tapparo et al. 2011, 2012, 2013; Girolami et al. 2009). It can be hypothesised that the foragers (and/or the workers in the hive) possess an advantageous sensitivity to toxic substances considering the mode by which they avoid introducing poisons into the hive before they can reach concentrations dangerous to the queen, young bees, and the brood.

## Data Availability

All the experimental results are reported in this article. Detailed experimental data can be provided upon request.
